# Exploring the general public’s and experts’ risk and benefit perceptions of cultured meat in Singapore: A mental models approach

**DOI:** 10.1371/journal.pone.0295265

**Published:** 2023-11-30

**Authors:** Shirley S. Ho, Mengxue Ou, Zhing Ting Ong

**Affiliations:** 1 Wee Kim Wee School of Communication and Information, Nanyang Technological University, Singapore, Singapore; 2 School of Social Sciences, Nanyang Technological University, Singapore, Singapore; National Healthcare Group, SINGAPORE

## Abstract

Despite the recent approval of cultured meat products in Singapore, the understanding of public perceptions towards this novel food technology remains limited. Utilizing attitude formation theory and the mental models approach, this study compares the mental models of the general public and experts regarding their risk and benefit perceptions of cultured meat. Through four online focus group discussions with 40 participants, we found convergences in the mental models of experts and the general public concerning perceived individual- and societal-level benefits of cultured meat (e.g., health benefits and food security) as well as their perceived individual-level risks of cultured meat (e.g., potential health issues and affordability). However, divergences in understanding societal-level risks were noted; the public expressed concerns about the challenges of cultured meat to religious and racial dietary customs, while experts highlighted potential investment uncertainties due to unclear consumer acceptance of cultured meat. Theoretical and practical implications are discussed.

## Introduction

With the global demand for livestock products, such as meat, continuously rising, there is an increasing need to address the environmental and ethical concerns associated with the traditional livestock industry [1]. Cultured meat, also known as lab-grown or cell-based meat, has emerged in response to these challenges as a potential solution to mitigate the environmental, ethical, and human health impacts of traditional livestock farming [2]. As a country heavily reliant on imported food, Singapore has actively promoted the development of novel food technology industries, including cultured meat, to enhance its self-sufficiency in food production [3]. Consequently, the market for novel foods, including cultured meat, is expanding, enticing more companies to enter the field [4]. Given the rapid growth of the cultured meat industry, a comprehensive understanding of consumer perceptions towards cultured meat is needed [5].

Previous research underscores the significance of individuals’ perceptions regarding the risks and benefits associated with novel food products, as these perceptions determine their acceptance or rejection of such products [5–7]. Concerns about the health and safety risks of novel food products have emerged as the primary factor contributing to their rejection [6]. Conversely, positive perceptions of benefits have been shown to enhance the acceptance of novel foods [8]. Furthermore, Verbeke et al. [9] found that while consumers recognized the societal benefits of cultured meat, they anticipated limited direct personal benefits, indicating a divergence in the perception of benefits at societal and personal levels. Additionally, previous studies have unveiled discrepancies between experts and laypersons in their risk perceptions [10], highlighting the importance of distinguishing between experts’ and laypersons’ perspectives on the risks and benefits of cultured meat. Despite the importance of understanding public perceptions of the risks and benefits of cultured meat for promoting understanding of cultured meat, studies have yet to explore these perceptions at both personal and societal levels in non-Western contexts like Singapore, which is the first country to approve the commercial sale of cultured meat products. Thus, this study aims to examine the personal and societal risk and benefit perceptions of cultured meat among experts and laypersons in Singapore.

This study has two aims. First, informed by the attitude formation theory [11], which proposes that people’s attitudes can be formed at either a personal or a societal level [12], this study seeks to explore how the general public and experts perceive the risks and benefits of cultured meat from both individual and societal perspectives. Second, utilizing the mental model approach, which investigates the cognitive structures individuals employ to comprehend the world [13], this study aims to compare the mental models of the general public with those of experts, identifying the similarities and differences in laypersons’ and experts’ perceptions of cultured meat. Insights from this study will identify key risk concerns, benefits perceptions, and potential misperceptions that influence the public’s acceptance or rejection of cultured meat. This knowledge can subsequently be applied to formulate effective communication strategies designed to enhance consumer understanding of cultured meat.

## Literature review

### Study context

Cultured meat production has become feasible due to advancements in tissue engineering and biotechnology [14]. To produce cultured meat, cells are extracted from animals and cultured in suitable mediums, enabling them to multiply and develop into an end-product resembling minced meat [15]. Notably, Singapore has become the first country to approve cultured meat [16]. The government invested more than S$250 million in the food technology industry in 2020 and 2021. Additionally, the Singapore Food Agency (SFA), a regulatory authority tasked with ensuring food safety and security in Singapore [17], has introduced regulatory frameworks for cultured meat, marking Singapore as a pioneer in legislating these new food products [3]. Despite these advancements, there is a paucity of studies specifically exploring public perceptions of cultured meat in Singapore [18]. Consequently, this study aims to bridge this research gap by investigating how the general public and experts in Singapore perceive the risks and benefits of cultured meat.

### Attitude formation theory

Attitude Formation Theory (AFT) posits that attitudes can be formed through either a bottom-up or top-down process [11,12]. In the case of novel products or technologies, bottom-up attitude formation occurs when individuals base their attitudes on their personal perceptions of the product’s or technology’s advantages and disadvantages at an individual level [12]. These attitudes arise from personal risk and benefit assessments associated with the product or technology [12]. In contrast, top-down attitude formation places an individual’s views about a specific novel food product or technology within a wider framework of overarching attitudes and values [19]. Within this context, overarching socio-political attitudes, such as the perceived risks or benefits of the technology to nature, society, and the environment, significantly influence individuals’ personal attitudes toward a particular novel product or technology.

AFT has been widely applied in studies examining consumer attitudes and acceptance of novel food technologies [12]. A focus group discussion by de Barcellos et al. [12] revealed that consumer acceptance of novel food technology depends on their perceptions of its risks and benefits at both personal and societal levels. Similarly, a study by Nielsen et al. [19] showed that individuals’ perceptions of novel food technology are influenced by their general socio-political attitudes/values as well as their personal evaluations of the risks and benefits, which is in line with AFT’s assertion that attitudes can be formed through both bottom-up and top-down processes. Considering the impact of consumer attitudes on the acceptance of novel food technologies like cultured meat, it is crucial to explore how individuals perceive the risks and benefits of cultured meat from both individual and societal perspectives [19]. Consequently, this study utilizes AFT as a theoretical framework to investigate Singaporeans’ perceptions of the risks and benefits associated with cultured meat.

### Perceived risks of cultured meat

#### Perceived societal risks

Perceived societal risks associated with cultured meat primarily revolve around the potential negative consequences that the transition from conventional to cultured meat could have on society [9,20]. Previous research has highlighted concerns about the impact of cultured meat on societal organizations, cultural practices, and the environment. For instance, there are questions about how the adoption of cultured meat might affect social events, such as barbecues or Sunday roasts [9]. Moreover, some studies argue that the industrial-level production of cultured meat could potentially displace traditional farming practices and agricultural jobs [21]. These concerns extend to the future of farm animals, particularly if they are no longer needed for food production [21]. Additionally, while the cultured meat industry requires less land and emits fewer greenhouse gases than traditional livestock farming, numerous scientists have expressed concerns about environmental risks, arguing that cultured meat production could have a higher carbon footprint, especially when using conventional energy sources [22]. In light of the risk perceptions identified in previous studies, this research aims to understand the societal risk perceptions related to the consumption of cultured meat in the specific context of Singapore.

#### Perceived personal risks

Individuals often associate perceived personal risks of cultured meat with its unknown adverse health effects, such as potential nutritional deficiencies from long-term consumption [3,19]. Participants in various studies have expressed concerns that negative health effects from consuming cultured meat might only manifest after extended periods [9], leading to fear responses and diminished acceptance of such novel food technologies. Furthermore, the perceived "unnaturalness" and safety concerns regarding cultured meat serve as significant barriers to its consumption [1]. Therefore, this study aims to glean insights into the personal risk perceptions of cultured meat among individuals in Singapore. Understanding these perceptions enables further exploration of the factors influencing the acceptance or rejection of cultured meat at a personal level.

### Perceived benefits of cultured meat

#### Perceived societal benefits

The production of cultured meat is often perceived as offering substantial animal welfare and environmental benefits by avoiding large-scale animal slaughter [21]. Consumers also recognize its potential to contribute to reduced greenhouse gas emissions compared to conventional meat production through agricultural farming [23]. Furthermore, some studies have suggested that cultured meat could meet meat demand, alleviate global poverty, and enhance global food security and public health [1]. However, these societal benefits of cultured meat hinge on the assumption that its production can be scaled up and made affordable to the public [1]. Therefore, this study aims to investigate individuals’ perceptions of the societal benefits of cultured meat in the specific context of Singapore, providing valuable insights into how the technology is perceived and its potential societal implications.

#### Perceived personal benefits

Consumers have expressed several potential individual-level benefits associated with cultured meat consumption [9]. These perceived personal benefits include potential reductions in fat content and increases in nutritional value, which are viewed as advantageous for personal health outcomes [3,19]. However, while incorporating additives and properties (e.g., nutrients) into cultured meat might enhance its health benefits, it could also diminish consumer acceptance and amplify the environmental footprint of the entire production process [24]. Given the lack of transparency in the cultured meat production process, accurately gauging the nutritional values of currently available cultured meat on the market remains challenging [24]. Moreover, consumers perceive cultured meat as having a lower risk of zoonotic or food-borne diseases due to the controlled lab environment and reduced human-animal contact compared to conventional meat [25]. As the perceived personal benefits of cultured meat among Singaporean consumers are still unclear, this study aims to explore perceptions of personal benefits associated with cultured meat in the Singapore context, providing insights into how individuals perceive its potential advantages at the individual level.

#### Mental model approach

Mental models are cognitive frameworks used by individuals to process information, interpret the environment, make predictions, and solve problems [13]. Individuals’ mental model reflects their internal representation of concepts and relationships in the real world [13]. When faced with uncertainty, people construct mental models based on their interpretation of the environment, and individuals with similar background or experiences tend to have similar or convergent mental models [13].

Research on mental models has primarily focused on understanding individuals’ cognitive processes, beliefs, and ideas that consciously or unconsciously form based on their experiences. Analyzing mental models can provide valuable insights for risk communicators, enabling them to assess the target audience’s level of knowledge and develop more effective and targeted risk communication strategies [26]. For example, a survey study examining the understanding of climate change among educated laypeople in the United States revealed the presence of problematic misconceptions in their comprehension of climate change. The study emphasized the importance of differentiating between "weather" and "climate" in news reports and political commentary to address these misconceptions and enhance public understanding [27]. Another study by Byram et al. [28] focused on the risk perceptions of women with breast implants regarding local complications. The research identified gaps in the participants’ mental models of their risk perceptions related to breast implants, which hindered their decision-making process. This finding highlights the significance of addressing and clarifying misconceptions or gaps in mental models to support informed decision-making [28].

However, limited research has delved into the mental models of various stakeholders (i.e., the general public vs. experts) regarding the perceived risks and benefits of cultured meat on both personal and societal levels. While existing research [29] has shown that individuals with different demographic traits (e.g., age) would exhibit varying acceptance rates of cultured meat, there is a dearth of studies comparing how the mental models of the general public, in terms of their risk and benefit perceptions of cultured meat, differ from (or are similar to) those of experts. Given that individuals with different backgrounds and expertise may hold distinct perceptions and attitudes towards cultured meat, investigating the mental models of different stakeholders can enhance our understanding of their risk and benefit perceptions of cultured meat at both individual and societal levels [13]. Therefore, drawing upon the mental model approach and attitude formation theory, this study aims to examine the mental models of different stakeholders regarding the perceived personal and societal risks and benefits of cultured meat. Accordingly, we propose the following research questions:

RQ1: What are the mental models of the general public and experts regarding their perceived personal and societal risks of cultured meat?
RQ2: What are the mental models of the general public and experts regarding their perceived personal and societal benefits of cultured meat?

### The lay-expert divide: A comparison of mental models

The mental models of the general public and experts often differ in terms of their understanding and interpretation of various issues, including environmental and health hazards, as well as food risk management [30,31]. Numerous studies have utilized the mental model approach to examine disparities in risk perceptions between experts and the general public on issues such as climate change and vaccines [26]. For instance, a study by Byram et al. [28] examining perceptions of local complications related to breast implants found that women’s beliefs about their personal risk of complications often diverged from those of medical specialists (i.e., experts) when comparing their mental models. Hagemann and Scholderer [26] also conducted a study comparing the mental models of experts and laypersons regarding mutation-bred rice, a type of rice varieties that have been developed through the process of induced mutation that involves the deliberate exposure of rice seeds or plant tissues to radiation or chemicals to induce genetic changes or mutations [32]. They found that experts were more focused on risks assessable within legal frameworks, whereas consumers expressed other concerns, such as environmental risks that fell outside these frameworks. These differences in mental models between laypeople and experts can result in communication gaps between them, hampering the effectiveness of risk communication [26]. Therefore, to better understand the disparities in mental models between experts and the general public regarding cultured meat, our study raises the following question:

RQ3: How do the general public’s and experts’ mental models compare?

## Methods

We conducted four online focus group discussions (FGDs) with the general public and cultured meat experts separately. FGDs are suitable for exploring perceptions as they allow for participant-led discussions and group interactions that can evoke attitudes or opinions regarding a certain topic, such as cultured meat, which might be difficult to elicit organically in a one-to-one interview [33]. Moreover, the exchange and comparison of opinions in a group setting can provide deeper insights than the cumulative findings from individual interviews [34]. This study employed online FGDs as they offered greater convenience for participants who had constraints in terms of time and location, allowing us to expand the pool of potential participants and capture a wider variety of perspectives [35]. Additionally, as the study was conducted during the COVID-19 pandemic, the online nature of this study also aligned with local guidelines on safe distancing in Singapore.

### Ethics statement

This study received approval from the Institutional Review Board (IRB) of Nanyang Technological University in Singapore (IRB number: IRB-2021-998). Prior to participating in the study, all participants provided their electronic consent by selecting “Yes, I am willing to participate in this study” in an online survey.

### Sampling and recruitment

We conducted four online focus groups–three with participants from the public and one with cultured meat experts–in Singapore from February to April 2022. The group sizes ranged from nine to eleven participants. Each session lasted approximately 2 hours, and participants were compensated with S$50 at the end of each session.

For the focus groups conducted with the general public, participants were recruited using a combination of convenience and snowball sampling methods. Recruiters utilized various techniques, including word-of-mouth, posters, and social media to reach potential participants. A total of 29 general public participants, who were not experts in novel food technology or cultured meat, were recruited. Only Singaporeans or permanent residents over 21 years old were included. Education levels of the participants ranged from The Singapore-Cambridge General Certificate of Education Normal Level (GCE N-level) to Master’s degree, with a majority holding a bachelor’s degree. The Singapore-Cambridge General Certificate of Education Normal Level (GCE N-Level) is an educational qualification attained upon successfully passing the Singapore-Cambridge General Certificate of Education Normal Level examination in Singapore [36]. To ensure more active participation, Krueger [37] suggested that discussion group participants share similar characteristics, such as age, gender, and ethnicity. Thus, participants were categorized based on the generational definitions provided by the Pew Research Center, which include Millennials (aged 21–41), Generation X (aged 42–57), and Baby Boomers (aged 58–75).

On the other hand, experts in the field of cultured meat and novel food technologies were identified using publicly available information from their affiliated institutions (e.g., universities and research institutions). Experts in the field of cultured meat and novel foods were either faculty members from academia or scientists from research institutes, specializing in various areas related to cultured meat and novel food technology, such as cell-based meat, stem cell biology, cellular agriculture, tissue engineering, culture medium, and bioreactor processes. We contacted and recruited experts via publicly available email and telephone contacts. In total, 11 scientists from research institutes and assistant professors (and above) from academia were recruited. Furthermore, all participants in this study were required to be comfortable using the online teleconferencing platform, Zoom. The demographic characteristics of the focus group participants are presented in Table 1.

**Table 1:** Details of online focus group participants.

Focus Group	Participants (No. and Gender)	Age	Age Group	Median of Education Levels
FGD1	10 (5 males, 5 females)	21–38	Millennials	Bachelor’s Degree
FGD2	10 (5 males, 5 females)	43–56	Generation X	Diploma & Professional Qualification
FGD3	9 (4 males, 5 females)	58–73	Baby Boomers	Bachelor’s Degree
Experts	11 (7 males, 4 females)	29–73	——	Doctoral degree

*Note*. The age groups were categorized based on the definition of Pew Research Centre [38].

### Moderation and guide

The online FGDs were conducted in English, the lingua franca of Singapore. Lingua franca is a language used for communication between groups of people who speak different languages [39]. A moderator from the research team facilitated the sessions with the help of assistant moderators. FGD 1 to FGD 3 were moderated by a doctoral student well-trained in social science research, while FGD 4 was moderated by a college faculty member in the field of social science with FGD moderation experience. A semi-structured moderator’s guide was developed based on the research aims of this study. The guide consists of a list of questions and prompts to gauge participants’ perceptions of cultured meat. First, participants were asked to introduce themselves. Participants were then asked about their general awareness of cultured meat. This question was not raised in the experts FGD due to their expertise on cultured meat. Subsequently, key questions were raised about their risk and benefit perceptions of cultured meat at both the personal and societal levels (e.g., “Do you think cultured meat has any potential benefits for Singapore society?”). Apart from the questions concerning the risk and benefit perceptions of cultured meat, the moderator’s guide also contained other questions, such as the information sources used by the participants to acquire information on cultured meat and their trust in these information sources. However, these questions are not the focus of this study.

### Analysis

The online FGDs were digitally recorded and transcribed verbatim. All personal identifiers were removed to ensure participants’ anonymity and confidentiality. Participants are labelled with numbers to maintain anonymity. For instance, G1P1 refers to Participant 1 from the General Public group 1 and EGP2 refers to Participant 2 from the Expert group. Each transcript was coded and analyzed by two coders who were members of the research team. A hybrid coding approach was used, combining both inductive and deductive approaches to analyze the data. This approach allows for the identification of data relevant to the research question through deductive coding, while also allowing for new themes to emerge directly from the data through inductive coding [40]. During the inductive coding process, the two coders independently read the transcripts line-by-line and generate codes by considering their relevance to the research questions. Each code was compared to the existing codes to determine if a new code was needed or if the previous codes needed revision. In the deductive coding process, the codes were categorized into primary conceptual themes in line with the research questions, i.e., personal benefits, societal benefits, personal risks, and societal risks. During the coding process, the two coders analyzed the transcript and generated codes independent of each other. The codes generated were then compared and modified in an iterative process to reflect the data collected, the relevance to literature, and the research scope.

## Results

### Mental models: Risk and benefit perceptions of cultured meat

**Fig 1:**
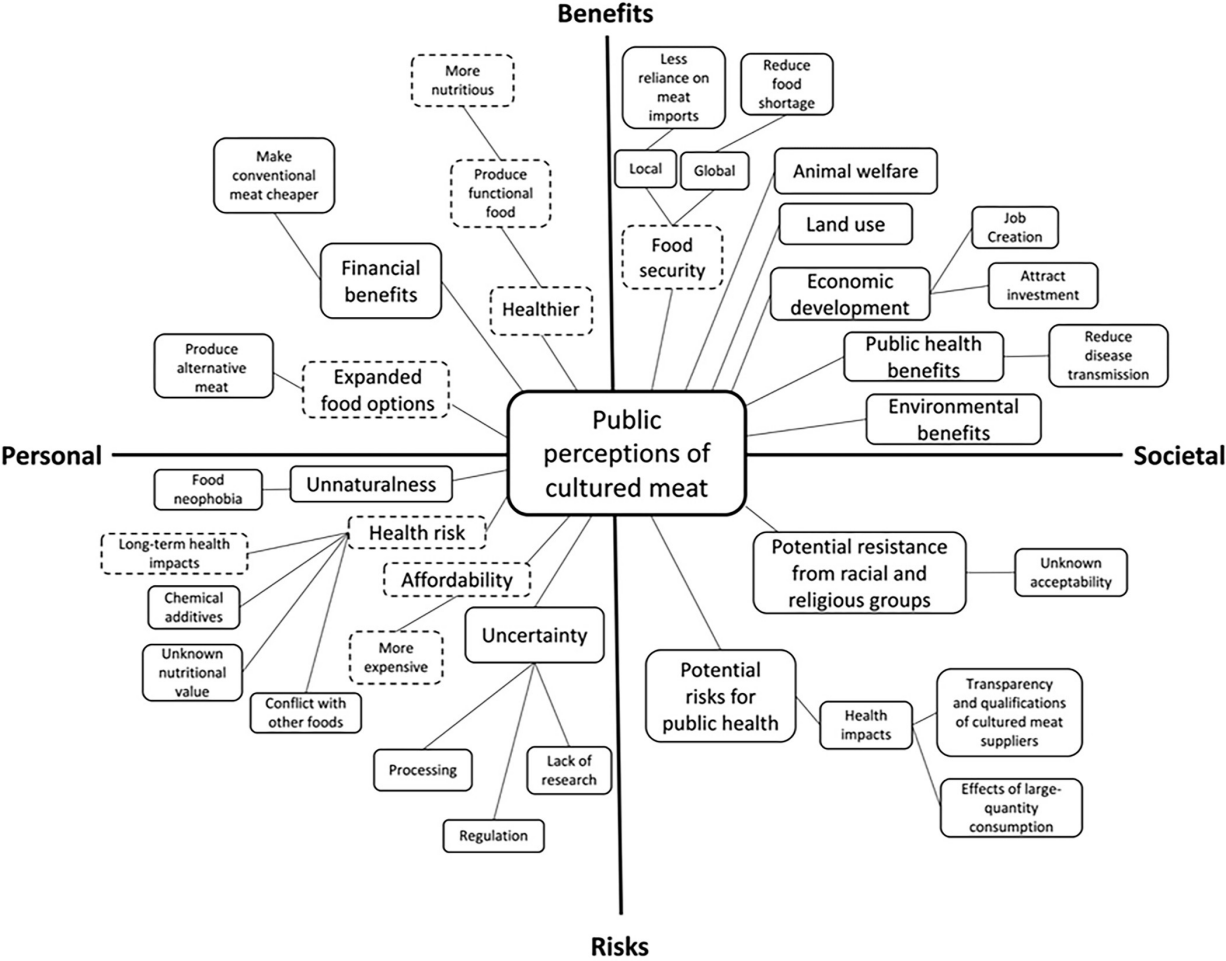
The general publics’ mental models regarding their risk and benefit perceptions of cultured meat. The dashed border refers to the area of convergence between the mental models of the general public and experts.

**Fig 2:**
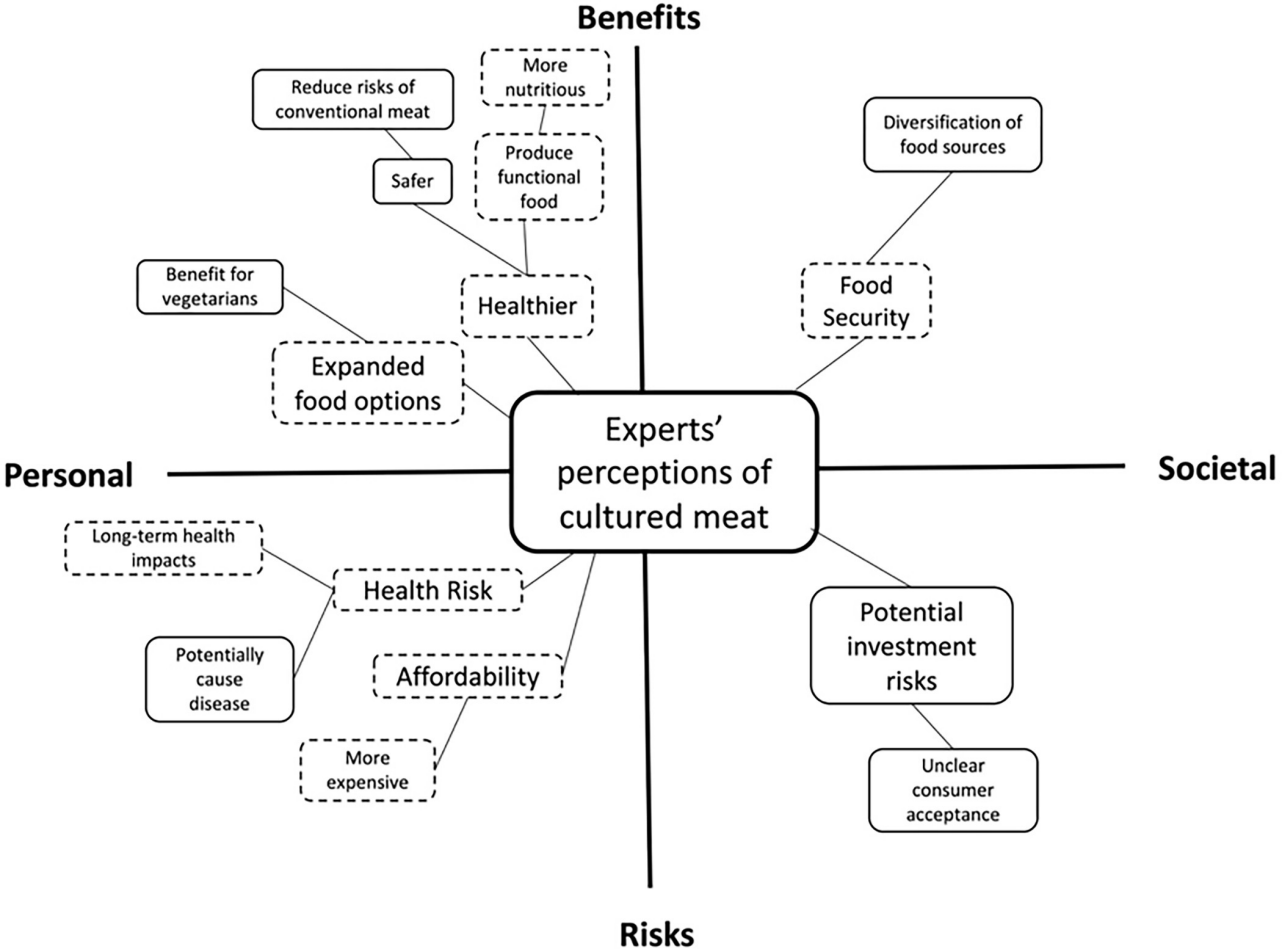
Experts’ mental models regarding their risk and benefit perceptions of cultured meat. The dashed border refers to the area of convergence between the mental models of the general public and experts.

The general public and experts discussed a wide range of personal and societal risk and benefit perceptions of cultured meat. Public perceptions of personal benefits included improvements in personal health, personal financial benefits, and expanded food options, while societal benefits encompassed food security and environmental benefits. On the other hand, public perceptions of personal risks involved concerns about personal health and affordability, while societal risks included public health risks as well as concerns related to race and religion. Experts had similar perceptions of personal and societal risks, albeit with differences in the level of comprehensiveness. Figs 1 and 2 provide visual representations of the mental models of the public and the experts, respectively. illustrating their risk and benefit perceptions of cultured meat at personal and societal levels.

### Public’s mental models of personal benefit perceptions

The mental models of the general public regarding their perceived personal benefits of cultured meat were health benefits, economic benefits, and expanded food options (see Fig 1).

### Perceived benefits for personal health

Generally, the general public participants felt that cultured meat could have benefits for their personal health. During the FGDs, participants discussed two main aspects of personal health benefits that cultured meat could offer–functional foods and food safety. In terms of functional foods, some participants highlighted that cultured meat could be engineered to be more nutritious and healthier (i.e., more proteins or minerals, less fat, or calories) than conventional meat (e.g., G1P6 & G1P7). Regarding food safety, some participants mentioned that because cultured meat is produced in the lab, it is expected to be cleaner than conventional meat and carries a lower risk of transmitting zoonotic diseases.

### Perceived expanded food options

Expanded food options were also perceived as a benefit of cultured meat. The general public participants believed that cultured meat could provide more choices for individuals as an alternative to conventional meat. For example, one participant (G2P2) expressed that cultured meat offered eco-conscious individuals the opportunity to select a more environmentally friendly option compared to traditional meat. The availability of diverse food options was seen as a personal benefit of cultured meat.

### Perceived personal financial benefits

The positive impact of cultured meat on individuals’ financial well-being was also perceived to be a personal benefit that cultured meat could provide. One participant (i.e., G1P8) noted that cultured meat could potentially “make the prices of meat lower” by competing with conventional meat in the market. Another participant (G1P4) considered the possibility that cultured meat could be more affordable than conventional meat if the lab production process becomes cheaper than traditional livestock rearing. Overall, participants believed that cultured meat could provide financial benefits as it has the potential to drive down the prices of other meat options.

### Public’s mental models of societal benefit perceptions

The mental models of the general public regarding the perceived societal benefits of cultured meat encompassed several key aspects. These included benefits for food security, the national economy, land use, public health, the environment, and animal welfare (see Fig 1).

### Perceived benefits for food security

The majority of participants acknowledged that a key societal benefit of cultured meat is food security. Given Singapore’s reliance on imported food, they suggested that cultured meat could enable the country to "grow our own meat" (G1P5), thereby increasing self-sufficiency, diversifying food sources, and mitigating supply chain vulnerabilities. Furthermore, in light of the growing global population, one participant noted that cultured meat has the potential to "address the issue of global food shortages" and "combat malnutrition" in developing countries (G3P4).

### Perceived benefits for Singapore economy

One of the key societal benefits of cultured meat discussed by the general public participants is its positive impact on the Singaporean economy. Three aspects of economic benefits were mentioned–reducing meat imports, increasing foreign direct investments, and creating more jobs. Participants (e.g., G1P5) mentioned that cultured meat could be advantageous for the economy as it would enable Singapore to enhance its self-sufficiency in meat production, thereby reducing the money spent on importing meat. Additionally, participants (e.g., G2P1) believed cultured meat, being an innovative food technology, would make Singapore more appealing to foreign investors who are interested in investing in such technologies and innovations. Moreover, participants (e.g., G3P2) also highlighted that the growth of the cultured meat industry could generate more job opportunities in Singapore, which would have a positive impact on the economy.

### Perceived benefits for land use

During discussions on the societal benefits of cultured meat, participants highlighted land use efficiency as a significant advantage. They pointed out Singapore’s land scarcity challenge and recognized that cultured meat could alleviate this issue due to its lower land requirements compared to conventional meat production. One participant noted, "It doesn’t really need to have animal farms" (G2P5), while another underscored the importance of alternative food production methods due to Singapore’s limited land and natural resources (G2P3). Both acknowledged the potential of cultured meat to effectively address land constraints, marking it as a substantial societal benefit.

### Perceived benefits for public health

Improving public health was also seen as a significant societal benefit of cultured meat, as discussed in focus groups with the general public. One participant noted that cultured meat could reduce the need for “interactions between animals and humans” (G2P5), potentially lowering the risk of zoonotic diseases like COVID-19. Another suggested that cultured meat might be cleaner than traditional meat, thus diminishing the risk of foodborne illnesses such as salmonella. Collectively, participants acknowledged the potential of cultured meat to enhance public health outcomes.

### Perceived benefits for the environment

The general public perceived cultured meat as an environmentally friendlier alternative to conventional meat and considered this to be a societal benefit. Some participants showed awareness of the environmental challenges faced by the livestock industry and perceived cultured meat to be more beneficial for the environment. For example, one participant acknowledged the environmental challenges posed by raising livestock for human consumption, stating that "having livestock for human consumption is an environmental challenge" (G2P4). Similarly, another participant believed cultured meat would contribute to a cleaner environment for society, mentioning that if "we don’t keep all these livestock, our environment is actually cleaner” (G2P2). These insights reflect the public’s perception of the positive environmental impact of cultured meat.

### Perceived benefits for animal welfare

Participants also recognized animal welfare as a benefit of cultured meat from a moral standpoint. The production of cultured meat, which eliminates the need for animal slaughter, was perceived by one participant as a more humane approach compared to the production of conventional meat (G3P4). Another participant shared this viewpoint, suggesting that cultured meat could impact the production and rearing of animals, ultimately reducing the necessity for animal killing (G3P3). These perspectives highlight the potential of cultured meat to address ethical concerns related to animal welfare.

### Public’s mental models of personal risk perceptions

The mental models of the general public encompassed several perceived personal risks associated with cultured meat, including personal health risks, uncertainties surrounding the technology, potential affordability issues, and the unnaturalness of cultured meat (see Fig 1).

### Perceived risks for personal health

When considering the health risks associated with cultured meat, many participants expressed concerns about its potential long-term effects on the human body. Some participants were also apprehensive about the presence of preservatives and chemical additives, perceiving them as potential health risks. For instance, one participant stated, "Cultured meat would have a lot of chemicals inside, which I don’t know what they are and what they might do to my body (G2P1)". Other participants shared similar concerns and doubts regarding the nutritional value of cultured meat. Furthermore, participants raised concerns about potential conflicts with other foods and the impact on individual health, considering them as personal risks associated with cultured meat.

### Perceived uncertainty

Participants expressed their uncertainties regarding cultured meat in various aspects, such as the science behind it, its processing methods, and the regulations governing its production. These uncertainties were considered personal risks by the participants. For instance, participants (e.g., G3P3) were unsure about the specific ingredients used in cultured meat and expressed concerns about the lack of long-term research on its potential side effects. Additionally, participants (e.g., G3P6) were uncertain about how to handle and cook cultured meat, as they were unsure if the same methods used for conventional meat would apply. Furthermore, participants voiced uncertainties about the regulatory framework surrounding cultured meat and questioned which government agencies would be responsible in case of any issues or mishaps, perceiving this as a risk associated with cultured meat.

### Affordability concerns

Affordability emerged as a major concern among participants discussing cultured meat. Since consumers bear the costs of their consumption, many identified the price of cultured meat as the primary factor influencing their purchasing decisions. For instance, one participant stated, "If it’s a cheaper option, I would definitely consider buying it. However, I think I would not pay a premium for lab-grown meat" (G1P9). Others also voiced concerns about the perceived higher cost of cultured meat compared to conventional meat. They saw the prospect of consuming expensive cultured meat as a financial risk, which could deter their willingness to consume it.

### Perceived unnaturalness

During the discussion on personal risks, the general public participants also expressed concerns regarding the unnaturalness of cultured meat. They perceived cultured meat as unnatural and noted that there would be "psychological resistance (G1P3) " towards consuming it. Aversion towards consuming cultured meat was considered as a personal risk associated with cultured meat. Additionally, the different taste or texture of cultured meat compared to conventional meat was also mentioned as a concern when discussing the unnaturalness of cultured meat. Some participants mentioned that as cultured meat is produced in a lab, it may not be as fresh as conventional meat and could have different tastes or textures. Such perceptions were regarded as risks associated with cultured meat that could influence individuals’ willingness to consume it.

### Public’s mental model of societal risk perceptions

Regarding the perceived societal-level risks of cultured meat, the FGDs among the general public identified several key themes. These included public health risks and potential resistance from certain racial and religious communities (see Fig 1).

### Perceived risks for public health

Health risks for the general public were highlighted as another potential societal risk associated with cultured meat during the discussions. Participants expressed concerns about the long-term effects of cultured meat on human health, considering it as a societal-level concern. Transparency and qualifications of cultured meat suppliers were raised as an issue, with participants expressing worries that suppliers might conceal potential side effects on human health. Moreover, participants expressed concerns about the societal consequences of consuming large quantities of cultured meat, drawing a parallel with diet soda where adverse side effects become evident only with significant consumption (G3P8).

### Potential resistance from certain racial and religious communities

During the focus group discussions (FGDs), potential resistance from certain racial and religious communities (e.g., Muslims) were identified as societal risks associated with cultured meat. Participants highlighted that certain racial and religious groups in Singapore (e.g., Muslims who adhere to strict dietary restrictions) may find it challenging to accept cultured meat, perceiving this as a societal risk. They also emphasized the need for approval or certification to enhance acceptance among diverse racial and religious communities. Transparency in the production process was another area of concern, as participants believed that lack of transparency could trigger negative reactions from these groups, potentially impacting society adversely. Overall, these discussions underscored the importance of addressing racial and religious concerns as integral factors in assessing the societal risks associated with the acceptance and adoption of cultured meat.

### Expert’s mental models of personal benefit perceptions

During the focus group discussion with cultured meat experts, participants identified two main personal benefits of cultured meat, including health benefits and increased food options (see Fig 2).

### Benefits for personal health

Experts considered cultured meat to have benefits for individuals’ health. They pointed out that it would be possible to improve cultured meat with certain bio-nutrients (E1P4), thereby making it healthier or more nutritious. Another expert (E1P7) shared his perspective on the personal health benefits of cultured meat, stating that it could help address the challenge of obtaining sufficient protein in his diet and improve his overall well-being. Additionally, experts highlighted that cultured meat could mitigate traditional risks associated with conventional meat, such as pesticide exposure, thus contributing to better personal health outcomes.

### Expanded food options

Increased food options were also identified as a benefit of cultured meat for individuals. Experts highlighted that cultured meat would expand consumers’ food choices and "diversify our diet” (EGP10). Furthermore, an expert who identified himself as a vegetarian emphasized that having cultured meat as an option would enable individuals like him to experience the flavors and textures of meat while still adhering to their dietary preferences.

### Expert’s mental models of societal benefit perceptions

When discussing the perceived societal benefits of cultured meat, the primary theme that emerged from the experts’ focus group discussion was food security (see Fig 2).

### Benefits for food security

Regarding the perceived societal benefits of cultured meat, the primary theme that emerged from the focus group discussion among experts was food security. Experts viewed food security as a significant advantage of cultured meat for Singaporean society. They highlighted that cultured meat could contribute to the diversification of food sources in Singapore and offer stability during disruptions in the supply chain. In situations where environmental or climate changes may lead to limitations in the availability of traditional food sources in Singapore, cultured meat could serve as a viable alternative, ensuring the continuity of food production. Overall, experts believed that cultured meat had the potential to enhance Singapore’s food security, making it a valuable benefit for society.

### Expert’s mental models of personal risk perceptions

Experts identified two main themes in terms of the risks of cultured meat on individuals: personal health risks and affordability concerns (see Fig 2).

### Potential risks for personal health

Experts raised concerns regarding the risk of cultured meat to personal health. Despite the current regulatory approval for some cultured meat products in Singapore, some experts acknowledged that the technology is still immature and believed that more long-term research on the impacts of cultured meat on the human body was needed. Other experts also highlighted potential hazards associated with cultured meat. For instance, an expert mentioned the possibility of certain animal proteins integrating into the human body and causing diseases. Additionally, experts generally agreed that more scientific research needs to be conducted to address these health risks.

### Affordability concerns

Affordability also emerged as a significant consideration in the discussion of personal risks during the FGD. From an industry perspective, experts expressed concerns regarding the affordability of cultured meat. If cultured meat were significantly more expensive than conventional meat, people would likely be reluctant to consider consuming it. This affordability issue was viewed as a potential barrier to the widespread adoption and acceptance of cultured meat by the general public.

### Expert’s mental models of societal risk perceptions

The focus group discussion among experts revealed one main theme regarding the societal risks of cultured meat, namely investment risks (see Fig 2).

### Investment risks

From the experts’ discussion, one main theme emerged regarding societal risks–investment risk. During the discussion, experts approached this topic from an industry perspective and expressed concerns about the potential risks to industry investments. They generally regarded the current landscape of cultured meat as "challenging” (EGP2) and a "commercial liability” (EGP6), given the limited acceptance of cultured meat among Singaporeans. One expert (i.e., E1P8) provided further insight by noting that vegetarians would not be inclined to consume cultured meat. The experts identified the risks to industry investments as a societal risk associated with cultured meat.

### Comparing the general public’s and experts’ mental models

The FGDs revealed convergences in the mental models of both the general public and experts regarding their perceptions of the personal and societal benefits and risks of cultured meat. Specifically, both the public and the experts perceived cultured meat to have personal health benefits, expand food options, and ensure societal food security. For example, participants from both the public and experts group believed that cultured meat would contribute to Singapore’s food security by diversifying food sources and reducing reliance on food imports. Additionally, both the general public and the experts expressed similar concerns about cultured meat in terms of its health risks and affordability. For example, participants from both groups were concerned about the long-term impact cultured meat could have on people’s personal health.

The mental models between the general public and the experts also exhibited some differences. In general, the general public perceived a broader range of societal-level risks and benefits compared to the experts. The experts focused more on the personal-level risks and benefits of cultured meat, while the general public had a more balanced perception of the risks and benefits of cultured meat across both personal and societal levels (see Figs 1 & 2). Additionally, the mental models of the experts had a relatively narrower focus on the risks and benefits of cultured meat across personal and societal levels, whereas the mental models of the general public had a more diversified and wider range of considerations. For example, when discussing the societal-level benefits and risks of cultured meat, the general public mentioned benefits for land use, animal welfare, and Singapore economy, as well as risks regarding public health and potential resistance from certain racial and religious communities, which were not mentioned by the experts.

Comparing the mental models of the general public and experts also exposed certain misconceptions about cultured meat among the general public. Particularly, some individuals in the general public groups mistakenly associated cultured meat with plant-based meat, which refers to alternative proteins derived from plants like soybeans. Additionally, while the general public perceived cultured meat as environmentally beneficial, experts still exhibited skepticism regarding these environmental benefits due to insufficient scientific evidence available in the existing literature (Chriki & Hocquette, 2020) [41].

## Discussion

This study aims to gain a better understanding of how individuals in Singapore, including the general public and experts, perceive the risks and benefits of cultured meat at both personal and societal levels. By examining their mental models, this study provides valuable insights into how individuals with different backgrounds and levels of expertise make sense of this emerging food technology. The nuanced examination of their perceptions can inform communication strategies and policies aimed at introducing cultured meat to the public. While previous studies have uncovered the public’s diversified considerations related to cultured meat (i.e., personal health benefits, animal welfare, and health risks) in the context of western countries [9], our results revealed interesting findings that are unique to the context of Singapore (i.e., benefits for land use and concerns from religious/racial groups). Additionally, the comparison between the public’s and experts’ mental models revealed differences in their evaluations of risks and benefits associated with cultured meat. Furthermore, our study identifies several misconceptions that may influence the public’s acceptance or rejection of cultured meat. These findings hold significant implications for public understanding and acceptance of cultured meat in Singapore.

### Risk and benefit considerations in the context of Singapore

This study has yielded intriguing findings concerning the perceptions of risks and benefits associated with cultured meat that are specific to the Singaporean context. These findings shed light on unique considerations that have not been extensively explored in previous studies on public perceptions of cultured meat., i.e., benefits for land use and risks related to the resistance from specific racial and religious groups.

### Potential resistance from racial and religious groups

When discussing the societal-level risks associated with cultured meat, participants expressed concerns about its impact on certain racial and religious groups in Singapore. They specifically raised questions about how different racial and religious groups, such as Muslims, would react to cultured meat. This aspect has not been extensively explored in previous studies on public perceptions of cultured meat. Singapore is a multi-racial and religious country, with a diverse population representing various races and religions [42]. Given that approximately 15% of the population in Singapore is Muslim, there exists a potential market for cultured meat consumption within this religious group. However, a key question for the Islamic community revolves around whether cultured meat aligns with Islamic dietary laws and should be classified as halal or not, based on Islamic principles. This presents challenges to the dietary practices and religious considerations [41] of Muslim groups. Previous studies have revealed that acceptance of cultured meat among Muslims is contingent upon the use of stem cells or tissues from halal sources and the meat being halal certified [35]. Recognizing these concerns from specific racial and religious groups, it would be valuable for future studies to explore the acceptance of cultured meat among different races and religious groups and understand the factors that shape their perceptions and attitudes towards this novel food technology.

### Benefits for land use

Our findings also revealed that the general public in Singapore perceived cultured meat to have significant benefits in terms of land use at the societal level. Participants believed that the adoption of cultured meat would free up valuable farmlands for alternative uses, such as residential, commercial, and transportation purposes. This perception of land use benefits associated with cultured meat is particularly unique in the context of Singapore, where land resources are limited and carefully managed due to the country’s unique circumstances and land constraints [43]. The emphasis placed by the Singapore government on prudent land use for sustainable development further underscores the relevance and importance of participants’ perceptions [44]. Our findings contribute valuable insights into the potential societal impacts of cultured meat in Singapore and provide a foundation for further research and policy considerations in this domain.

### Comparing the mental models of the general public and experts

The comparison between the mental models of the general public and experts revealed distinct differences in their approaches to perceiving the risks and benefits of cultured meat at both personal and societal levels. The mental model of the general public was found to be more comprehensive, holistic, and influenced by contextual factors, such as anecdotal information from others and Singapore’s unique characteristics (i.e., limited resources and a multi-racial society). On the other hand, the mental models of the experts were relatively narrower and more focused, drawing heavily on their topical knowledge and the existing scientific research on cultured meat. This divergence in the extensiveness of risk and benefit perceptions can be attributed to the disparities in relevant experiences and knowledge between the general public and experts. The general public’s perceptions were influenced by the societal contexts and personal experiences, whereas the experts relied more on their specialized knowledge and research findings in the field. This finding aligns with previous studies that have highlighted the differential perception of risks between laypersons and experts [10,31].

Additionally, the general public and experts demonstrated divergent perspectives on risk considerations. The public tended to lean towards avoiding risks altogether, as indicated by a participant’s comment (G1P3): “Maybe there’s an aversion to trying it or testing it because it’s genetically modified and it’s not organic, so I think for myself there would be a sort of psychological resistance against trying it.” In contrast, experts acknowledged the risks associated with cultured meat but viewed them as challenges to be addressed and managed in the future. Furthermore, the public expressed more uncertainty with regards to regulatory processes while the experts displayed greater trust in the regulatory system. These disparities in risk perception may stem from differences in the levels of trust placed in Singapore’s regulatory processes between experts and the general public. Experts, who have likely had direct experiences with Singapore’s robust regulatory system, are more likely to have higher levels of trust in the governing processes. These variations in risk considerations between the general public and experts highlight the importance of addressing public concerns and enhancing public trust in the regulatory framework.

### Implications

Theoretically, our study enhances the existing literature by scrutinizing individuals’ perceptions of risks and benefits related to cultured meat within the Singapore context. Though previous studies have conducted surveys to examine public perceptions of cultured meat in Asian countries [45], the utilization of focus group discussions in our study yielded more comprehensive information and elucidations regarding people’s interpretations of the risks and benefits of cultured meat. This study further categorizes perceptions of cultured meat risks and benefits into personal and societal dimensions, facilitating a more profound understanding of how perceptions regarding cultured meat risks and benefits are delineated across societal and individual levels. Previous research has found that laypersons and experts perceive risks differently in various domains, such as new food technologies and climate change [10,46]. In this study, comparing the mental models of laypersons and experts enabled us to explore how individuals, with varying degrees of topical knowledge about cultured meat, differ in their considerations of its risks and benefits, and how these differences are reflected in their perceptions.

Practically, discerning the primary risk and benefit perceptions the general public holds towards cultured meat can guide communication practitioners and policymakers in effectively conveying cultured meat information, thereby enhancing public understanding. Communication practitioners might address various risk concerns and emphasize the pertinent benefits of cultured meat in public dissemination. Moreover, our findings can refine public policies concerning cultured meat and novel foods. For example, as personal health risks emerged as a key public concern, policies could be crafted to assuage these specific health apprehensions. Notably, disparities between the mental models of the general public and experts revealed differing considerations regarding cultured meat’s risks and benefits at both personal and societal levels. Public perceptions often hinge more on contextual factors, such as Singapore’s limited land and resources and its multi-racial context, while expert perceptions lean towards their professional knowledge and research experiences. Consequently, policies and communication strategies should be grounded in these mental models, tailored to accommodate varying knowledge levels when communicating about cultured meat to the public.

Our study unveiled several misconceptions the general public holds regarding cultured meat, including confusion between cultured and plant-based meat, and an exaggeration of the environmental or health benefits of cultured meat (e.g., being more environmentally friendly and nutrient-rich). These misconceptions might be related to their personal knowledge about cultured meat and the current advertising from cultured meat companies, potentially influencing the general public’s attitudes and subsequent consumption intentions. Communication practitioners can crucially develop strategies to correct misconceptions related to cultured meat, ensuring the dissemination of accurate information. Collaborating with mass media channels and social media influencers can assist in reaching a broader audience and facilitating the spread of precise knowledge about cultured meat. Given that resources are finite, it is recommended to concentrate on debunking the identified misconceptions from this study, such as the confusion between cultured meat and plant-based meat. By specifically targeting these misconceptions, efforts can be directed to mitigate false beliefs among individuals unfamiliar with cultured meat.

### Limitations

Our study has some limitations. First, as the study is qualitative and exploratory in nature, findings cannot be generalized or replicated to a wider population or different cultural contexts. Further studies could be conducted to include a greater diversity of participants from different backgrounds or have an increased number of FGDs to allow for more meaningful comparisons (i.e., between people of different cultural backgrounds). Second, this study cannot draw statistical conclusions regarding causal relationships or correlations between individuals’ risk or benefit perceptions and their acceptance of cultured meat. As previous scholars suggested using the survey methodology to examine factors influencing the formation of mental models [47], future studies should attempt to employ quantitative research methods (e.g., survey) to examine the prevalence of beliefs, perceptions, and misperceptions regarding cultured meat as well as their impact on individuals’ mental models. Third, the narrower mental models of the experts than those of the general public may probably have much to do with the imbalanced number of participants in the FGDs between experts and the general public. Future studies could try to strike a balance between the number of participants from different stakeholders to gain more balanced and comprehensive insights from them.

## Conclusion

This study explores perceptions of risks and benefits associated with cultured meat among both the general public and experts, offering insights into Singaporeans’ unique perspectives, which include recognizing benefits related to land use and expressing concerns about resistance from specific racial and religious groups. While some convergence in the mental models of the public and experts was observed, differences in comprehensiveness and societal risk considerations were noted. The study also identified public misconceptions about cultured meat, underscoring the need to address these by utilizing communication strategies. The findings contribute to the development of effective strategies to promote public understanding of novel foods, including cultured meat, in Singapore. Future research could expand on these insights by exploring perceptions of cultured meat in other cultural contexts and incorporating quantitative methods to further understand contributors to people’s risk and benefit perceptions of cultured meat.
